# Study protocol: the effects of work-site exercise on the physical fitness and work-ability of older workers

**DOI:** 10.1186/1471-2474-8-9

**Published:** 2007-01-31

**Authors:** Martin Mackey, Chris G Maher, Terry Wong, Kathleen Collins

**Affiliations:** 1 Back Pain Research Group, School of Physiotherapy, The University of Sydney, PO Box 170 Lidcombe NSW, 1825, Australia; 2 Fit For Work Australia Pty Ltd, Sydney, Australia

## Abstract

**Background:**

Older workers have a higher rate and cost of injury than younger workers and with a rapidly ageing work force there is a need to identify strategies to address this problem. Older workers are less physically active and fit than younger workers and so have reduced work ability. The reduced work ability means they are more likely to be fatigued at work and so at greater risk of injury. Exercise could potentially assist this problem. Exercise training has been previously shown to improve fitness in older people however there has been no evaluation of workplace exercise program for older workers. We do not know if the programs are feasible and can improve the fitness and work ability of older workers. We have designed a randomised controlled trial to evaluate whether exercise improves fitness and perceived work-ability of older workers.

**Methods/Design:**

This paper describes the protocol for a trial examining the effects of a 12-week physical training program in workers over the age of 45. Participants will be randomized to an exercise or no-intervention control group. The primary outcomes are cardiorespiratory endurance, lifting capacity, upper and lower limb strength and perceived work-ability.

**Discussion:**

This trial will test the feasibility of implementing a worksite-based exercise program as a means of improving the physical fitness and work-ability of older workers performing physically demanding work. If we demonstrate the feasibility of the program we will conduct a larger trial that additionally measures injury outcomes.

## Background

The Australian population is ageing and this has significant economic and social implications. Currently, 82% of those aged 45–54 years are in the workforce. This participation rate declines to 55% in those aged 55–64 years. In the future employers will require a greater percentage of workers to remain in the workforce beyond the current retirement age to meet workforce demands. The Australian Bureau of Statistics [1] estimates that from 1994 to 2014, the 55–59 year age group will have the highest increase in workforce participation of all age groups.

**Figure 1:**
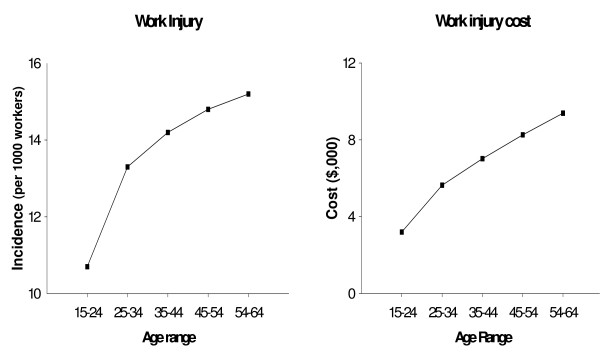
Relationship between (a) ageing and work injury incidence (left panel) and (b) ageing and injury cost (right panel). Figure created from data in [3].

The influence of an ageing workforce on occupational health and safety (OHS) has been of increasing concern to governments, industry and OHS regulators. This concern is evidenced by the recent publication of a 'Surveillance Alert on OHS and the Ageing Workforce [2]. There is a major problem with an ageing workforce; older workers are more likely to have a work injury than younger workers and their injuries are more costly than those of the younger workers [3] (Figure 1). For every 1,000 workers in New South Wales aged 15–24, 11 injuries per year would be expected, each costing $3,200 (median) or $35,200 in total. With the 54–64 age group, 15 injuries are expected, each costing $9,400 (median) or $143,000 in total. Thus Australia is facing a major public health challenge. There is an urgent need to identify strategies to encourage and enable older workers to remain in the workforce and at the same time to minimise the concurrent risk of work injury in this group. Amongst older workers sprain and strains to musculoskeletal tissues are the most common types of work injury with body stressing and slips, trips and falls the leading mechanisms of injury [4,5].

**Figure 2:**
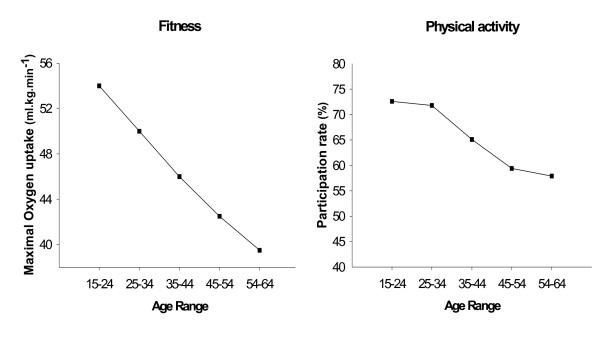
Relationship between (a) ageing and fitness (left panel) and (b) participation in sport and physical activit (right panel). Figure created from data in [8, 10].

When considering the OHS implications of the ageing workforce, it is recognised that ageing brings about a number of changes in physiological and cognitive abilities. For example, it is known that physical capacity (which includes fitness, muscle strength and flexibility) declines with age, and after 50 years of age this decline is more marked [6-8] (Figure 2). Age associated changes in the postural system also make it difficult for some individuals to adopt certain working positions [9].

However, in addition to naturally occurring physiological deterioration, the observed reduction in physical capacity with ageing may also be due, in part, to lower levels of physical activity in older individuals. In a recent survey, the Australian Bureau of Statistics [10] reported that participation in sport and physical activities substantially declines with age (Figure 2). The resultant reduction in fitness may reduce the ability of an individual to exert concentrated intense effort over a short period [11].

**Figure 3:**
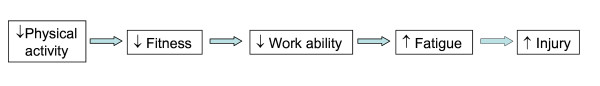
Conceptual model of ageing and injury.

Age-related decline in physical capacity is a major concern for workers involved in physically demanding work. If a worker's physical capacity cannot meet the task demands, it can cause excessive fatigue, leading to poor productivity along with an increased risk of industrial accidents [11] (Figure 3). It has also been consistently reported that physically demanding work does not maintain or improve physical capacity among ageing workers [8,12]. Compared to the younger workers, older workers need higher reserves for recovery and thus the ageing worker is more susceptible to fatigue and other adverse symptoms. This situation, together with the increased potential for chronic disease, decreases the safety margin which protects the worker against injury and work-related disease [13].

These findings concerning the OHS implications of an ageing workforce indicate that there is a need for interventions to maintain the work-ability of older workers. Potential interventions could be 'job-related' such as task modification, to ensure work demands are consistent with the reduced physical capacity of the ageing worker. Alternately interventions could be 'worker-related' such as exercise training, to improve the physical capacity of the ageing worker. Task-specific muscle endurance training and task modification have been shown to significantly increase work tolerance limits in healthy subjects performing a repetitive manual work task [14]. However, it is not known whether such interventions are applicable in older workers performing physically demanding work in industrial settings.

The purpose of this study is therefore to examine the feasibility of implementing a work-site based exercise intervention as a means of improving the physical fitness and work-ability of older workers performing physically demanding work. This project is a necessary first step to demonstrate 'proof of concept' in order to secure funds for a larger trial. This project will test response to treatment, treatment effect size and identify factors affecting adherence and compliance with treatment.

## Methods/Design

The University of Sydney Human Research Ethics Committee has granted approval for this study (Ref No: 12-2005/3/8798).

### Overview of research design

This study will be a randomized controlled trial of an exercise program for workers older than 45 year of age. The exercise program will consist of 36 individually supervised 40 minute sessions over a 12-week period with treatment outcomes measured at baseline and 12 weeks. The control group will receive no intervention.

### Randomisation

Participants will be allocated to treatment group using sealed opaque envelopes. The allocation sequence will be generated by author CM. Participants will be scheduled to receive their first treatment within one week of randomisation.

### Subjects

A minimum of 20 workers aged 45 years and over will be recruited with at least 10 subjects randomly allocated each to an experimental (exercise-training) group and a non-training control group.

### Screening

Potential participants will be screened for contraindications to exercise using the Physical Activity Readiness Questionnaire [15]. If a volunteer provides a positive response to items 1, 2, 3, 4, 6 or 7, the trial exercise physiologist will discuss the case with the chief investigators and if necessary a medical review will be undertaken to exclude any contraindication to exercise as listed in the ACSM guidelines [15].

### Treatments

The work-site exercise training intervention will include aerobic exercise (such as walking or cycling) and resistance training exercise to increase muscle strength and endurance. Subjects allocated to the exercise group will be encouraged to exercise 3 times per week for a maximum of 40 minutes each session during work time for 12 weeks. Following initial instruction subjects will exercise under supervision in a dedicated exercise space at the workplace. All subjects will be encouraged to continue with their normal leisure activity outside work. A full description of the exercise protocol is provided in the appendix [see additional file 1].

Participants randomized to the control group will be asked not to change their current activity levels or begin an exercise program.

### Outcome measures

#### Physical capacity

The following physical capacity tests will be taken at baseline and at the end of the intervention period.

- YMCA Cycle Ergometer [15]

- 1RM Bench Press [15]

- 1RM Leg Press [15]

- 80% of 1RM Bench Press [15]

- 80% of 1RM Leg Press [15]

- Sit-and-Reach [15]

- PILE Test (Lumbar) [16]

Subjects will perform the YMCA Cycle Ergometer test as a submaximal test of their cardiorespiratory endurance. The test results will be used to estimate maximal power output and energy expenditure, as indicators of aerobic fitness. Muscular strength will be assessed with the 1RM Bench Press and 1RM Leg Press, muscular endurance will be assessed as the number of repetitions of 80% of 1RM Bench Press and 80% of 1RM Leg Press. Low back and hip joint flexibility will be assessed using the sit-and-reach test. Lifting capacity will be assessed using the PILE test (lumbar protocol).

#### Work ability

The concept of work ability includes both the characteristics of the individuals themselves (their health and functional capacities; their competence, knowledge and skills; and their values, attitudes and motivations) and the characteristics of their working environment. Work ability will be evaluated before and after the intervention in all subjects by questionnaire using the Work Ability Index (WAI) [17,18]. The WAI includes 7 subjective estimations on work ability in the light of job demands and psychophysical resources, and also includes information about illnesses and work absenteeism.

#### Physical activity

The International Physical Activity Questionnaire IPAQ [19] will be used to measure subject's physical activity levels.

### Data analysis

We will use a regression model to test for the effect of treatment on outcome at follow-up with the baseline value of the outcome entered as a covariate. Alpha will be considered significant if it is less than 0.05.

## Discussion

We have presented the rationale and design of a randomized controlled clinical trial evaluating the effect of motor control exercise versus placebo in patients with chronic LBP. The results of this trial will be published as soon as they are available.

## Competing interests

TW is the principal of a business providing exercise-based rehabilitation programs for injured workers. KC is an employee of the business. Authors MM and CM declare that they have no competing interests.

## Authors' contributions

MM, CGM and TW were responsible for the design of the study. KC is responsible for implementing the exercise program and assessing outcomes. MM will act as trial coordinator. All authors have read and approved the final manuscript.

## Pre-publication history

The pre-publication history for this paper can be accessed here:



## Supplementary Material

Additional file 1 Appendix. Full description of the exercise protocol Click here for file
