# Supplementary material for: Study protocol: the effects of work-site exercise on the physical fitness and work-ability of older workers
Source: BMC Musculoskelet Disord. 2007 Jan 31;8:9. doi: 10.1186/1471-2474-8-9 (PMC1796542; doi:10.1186/1471-2474-8-9)
Supplement: Additional file 1 — Appendix. Full description of the exercise protocol [file 1471-2474-8-9-S1.doc]

**Appendix**

*Treatments*

After the participants have completed their baseline assessments they will then be randomly allocated to one of two groups; a control group that does not exercise but are to continue to perform their normal daily and work activities and an exercising group who exercise 3 times per week under supervision by the research assistant in a gymnasium set up at their workplace.

Considerations for exercise prescription:

- Exercise selection.

- Training frequency.

- Exercise order.

- Training load and repetitions.

- Volume.

- Rest periods.

- The exercise prescription will be restricted by the time in which the subjects have available to exercise in and the availability of exercise equipment.

The exercise sessions will include a warm up and cool down and the main exercise phase which will take the form of a circuit and involve aerobic and resistance exercises. The participants will be training for a maximum of 40 minutes however initial exercise time may be less due to their initial fitness levels.

The exercise group who exercise 3 times per week may have different exercise programs for each session. For example the participant may have two different training routines, routine 1 may be performed on training day 1 and training day 3 and routine 2 may be performed on training day 2. The specific exercise program for each participant will be determined after all exercise tests have been performed.

Exercise program:

**Warm up (5-10 minutes):**

- Will include 5 minutes of progressive aerobic exercise that will reach the lower limit of the prescribed heart rate for exercise (cycling or walking).

- 3-5 minutes of static stretches focusing on the major muscle groups (quadriceps, hamstrings, lower back, chest and upper back) and holding each stretch for up to 30 seconds.

**Exercise phase (20-30 minutes):**

Due to the limited time of this phase to perform both aerobic and resistance exercises they will be combined into a circuit.

The circuit will include >20 minutes of cardiovascular exercise and will also need to include upper and lower body strengthening exercise as well as an abdominal/lower back strengthening exercise.

An example of the possible prescribed circuit (excluding warm-up and cool-down):

- 7minutes bike/treadmill

- Leg Press: 1 set of 8-10RM

- Bench Press: 1 set of 8-10RM

- 7 minutes bike/treadmill

- 1 set of floor to waist lift 8-10RM

- 1 set of abdominal/lower back exercise

- 7 minutes stepper/bike/treadmill

- Rest time: will only be the time it takes to move between each exercise and setting up their station, such as changing weight, seat height etc. (~ 20-30 seconds).

- Exercise Order: As in the example program above exercises will be set out so as not to overload one muscle group in consecutive exercises. For example a leg strengthening exercise will then be followed by an upper body exercise.

- Exercise Selection: Will be based on training the testing apparatus (leg press, bench press and bike), include exercises that work the major muscles of the body and will also be work specific.

- For the resistance exercises a prescribed lifting repetition of 8-10RM will be used to maximize hypertrophy.

**Summary of prescription of exercises:**

Aerobic exercises:

- Available equipment will be a bike, treadmill and stepper.

- The circuit will involve the use of all of this equipment with the time being varied on each. For example:

(1) 3 x 7 minutes on bike, treadmill and stepper.

(2) 2 x 10 minutes on bike and treadmill.

(3) 1 x 15 minutes on bike or treadmill/ 1 x 5-7 minutes on stepper or bike.

(4) 1 x 20 minutes on bike or treadmill.

- Total aerobic training time will always add up to between 20-25 minutes due to time available to train. Greater aerobic benefits will be achieved by gradually increasing the intensity of the exercise as we are limited by increasing time.

*Establishing initial exercise intensity*:

- The Heart Rate Reserve method (Karvonen method) will be used to establish exercise intensity.

- Each participant will wear a heart rate monitor and the intensity of exercise will be gradually increased from between 50-85%HRR over the 12 weeks of training.

- Starting intensity will be ~50-60%HRR; however this may vary according to each individual’s initial level of fitness.

In this method the resting heart rate (HRrest) is subtracted from the maximal heart rate (HRmax) to obtain HRR. You then take 50% and 60% of the HRR and add each of these values to the resting HR to obtain the target HR range. This will be the target heart rate range to train between: Target HR range = ([HRmax – HRrest] x 0.50 and 0.60) + HRrest ((HRmax = 220 – age)

Resistance exercises:

- Resistance exercises will involve training the major muscle groups of the upper and lower body as well as the trunk muscles.

The following are examples of possible exercises:

*Lower Limb exercises:* Leg press, squats, lunges, walking lunges, step ups.

*Chest exercises:* Bench press, push ups, chest flys, dumbbell chest press.

*Upper Back:* One arm row, reverse flys, theraband 2 arm row.

*Arm exercises:* Bicep curl, tricep dips, theraband tricep pushdown, overhead shoulder press.

*Functional Lifting:* Floor to waist lift, floor to waist to chest lift, floor to waist to overhead lift, floor to overhead lift.

*Core Exercises:* Front hold, side hold, alternate arm and leg raises (lying or in 4-point kneeling).

*Establishing the initial workload:*

- After establishing each participant’s 1RM and with an initial work repetition set at 8-10 repetitions, a starting weight will be established from the 1RM.

- A relationship has been found between a sub-maximal load - calculated as a percentage of the 1RM – and the number of repetitions that can be performed at that load. Appendix Table 1 displays the percent of the 1RM and the amount of repetitions allowed:

- Training at 8-10 repetitions is training between 75-80% of the 1RM. Therefore in calculating 75% and 80% of each individual’s 1RM this will be their starting range.

- Adjustments can be made to assigned loads based on the observation of the ease or difficulty a participant experiences lifting the load for the required repetitions.

**Cool down (5 minutes):**

Will involve a gradual decrease in intensity of activities and include 5 minutes of warm down stretches the same as those prescribed for the warm up.

**Exercise progression protocol**

**Initial stage:** (1-4 weeks)

- Start at 50-60% HRR.

- Involve light muscle endurance exercises and moderate level aerobic activities.

- Duration of the exercise session may only last up to 15-20 minutes initially due to initial fitness levels.

Aerobic progression:

- Aerobic progression will be similar to that as recommended by ACSM [15] as outlined in Appendix Table 2.

- Depending on the results of the aerobic exercise test the participants will exercise between 50-60% HRR for the first two weeks.

- Progression for the first 4 weeks will be based on increasing the exercise duration for those that are unable to start at 40 minutes per session (involving aerobic and resistance exercises).

- As well as progressing exercise time as above they will also be progressed to training at 60-70% HRR for weeks 3-4.

Resistance progression:

A simple method that can be used to increase the participant’s training load is called the 2-for-2-rule.

*2-for-2-rule:* If the participant can perform 2 or more repetitions over their assigned repetitions in the last set in 2 consecutive workouts for a certain exercise, weight will be added to that exercise for the next session.

*Number of sets:* It has been shown that single-set training may be appropriate for untrained individuals, however many studies have indicated that further increases in volume are needed to cause further gains in strength.

It has been suggested that relative load increases of 2.5%-10% could be used to progress an individuals load lifted. Appendix Table3 provides an outline of possible load increases that could be implemented:

Overall, in the first 2 weeks the participants will perform one set for each exercise, if it is evident that they have adapted to this volume they will then be progressed to two sets for each exercise for weeks 3-4.

**Progress stage:** (5-12 weeks)

- Aerobic intensity should progress every 2-3 weeks.

- Our main form of progression for aerobic exercise will be through intensity as we are limited by progressing exercise time due to overall time available for each exercise session.

- Participants will eventually exercise at a moderate to vigorous intensity.

- Older and de-conditioned individuals may need more time at each stage to adapt to the training.

- The participants exercise diaries will be monitored regularly with the 2-for-2-rule and load increases being implemented throughout the program

Rates of progression are:

Week 5 & 6:

- Aerobic progression: 60-70% HRR

- Resistance progression: 2 sets of each exercise.

Week 7 & 8:

- Aerobic progression: 65-75% HRR

- Resistance Progression: 2 sets for each exercise.

Week 8 & 9:

- Aerobic progression: 70-80% HRR

- Resistance progression: 2 sets for each exercise.

Week 10 & 11:

- Aerobic progression: 70-80% HRR

- Resistance progression: 3 sets for each exercise.

Week 12:

- Aerobic progression: 75-85% HRR

- Resistance progression: 3 sets for each exercise.

Progression may be affected at various stages due to illness or slow adaptation to the training, intensity and volume progression will therefore be changed accordingly.

**Appendix Table 1. Relationship between load and repetiti**ons.

| **Percent of the 1RM and**  **Repetitions allowed** | |
| --- | --- |
| **(% 1RM-repetition relationship)** | |
| **%1RM** | **Number of repetitions allowed** |
| 100 | 1 |
| 95 | 2 |
| 93 | 3 |
| 90 | 4 |
| 87 | 5 |
| 85 | 6 |
| 83 | 7 |
| 80 | 8 |
| 77 | 9 |
| 75 | 10 |
| 70 | 11 |
| 67 | 12 |
| 65 | 15 |

**Appendix Table 2. Plan for progression of aerobic exercise.**

| **Program stage** | **Week** | **Exercise frequency sessions** | **Exercise intensity (%HRR)** | **Exercise duration**  **(min)** |
| --- | --- | --- | --- | --- |
| Initial stage | 1 | 3 | 40-50 | 15-20 |
|  | 2 | 3-4 | 40-50 | 20-25 |
|  | 3 | 3-4 | 50-60 | 20-25 |
|  | 4 | 3-4 | 50-60 | 25-30 |
| Improvement stage | 5-7 | 3-4 | 60-70 | 25-30 |
|  | 8-10 | 3-4 | 60-70 | 30-35 |
|  | 11-13 | 3-4 | 65-75 | 30-35 |
|  | 14-16 | 3-5 | 65-75 | 30-35 |
|  | 17-20 | 3-5 | 70-85 | 35-40 |
|  | 21-24 | 3-5 | 70-85 | 35-40 |
| Maintenance stage | 24+ | 3-5 | 70-85 | 30-45 |

**Appendix Table 3. Guideline for load progression with resistance exercise.**

| **Description of the participant** | **Body area**  **exercise** | **Estimated load increase** |
| --- | --- | --- |
| Smaller, weaker participant | upper body | 1-2 kg |
|  | lower body | 2-4 kg |
| Larger, stronger participant | upper body | 2-4+ kg |
|  | lower body | 4-7+ kg |
